# Cases of Diffuse Rheumatism

**Published:** 1826-07

**Authors:** 

**Affiliations:** St. George's Hospital.


### CASES OF RHEUMATISM.

Dr. Chambers is in the habit of distinguishing between those cases in which rheumatism is confined to the synovial bass and bursæ of tendons, and those in which all the neighbouring parts, which have a covering of fibrous membrane, become the primary seats of the disease. These varieties, though occasionally passing into each other, more frequently run a distinct and independent course,—the rheumatic affection occupying one or other of these textures exclusively. We subjoin a few cases which will serve to illustrate the characteristics and treatment of the *diffuse* form: in our next we shall present our readers with some examples of the *bursal* rheumatism.

*Cases of Diffuse Rheumatism.* Treated by Dr. Chambers, at St. George's Hospital.

I. May 17th, 1826.—Francis Hislop, ætat. twenty-two, baker, from St. Martin's-lane.

Complains of universal pain, which is however severest in the neighbourhood of the joints. The hands, wrists, and fore-arms are puffy and very painful, especially on the slightest motion. The feet and ankles are also similarly affected, though not to the same extent. There is no effusion into any of the synovial bags. Pulse 120, small and hard; skin dry and warm; tongue white; bowels open; urine high coloured. Ill ten days; was first attacked in the left leg and foot.

> Fiat VS. ad ℥xij. statim.

> Calomel, ℈ss.; Opii, gr. ij. omni nocte.

> Haust. Sennæ, omni manè.

> Diæta sit parcissima.

19th.—Blood highly inflamed; pain somewhat relieved; both wrists and hands are, however, much swelled and puffy. Pulse 110, full and soft; tongue white; skin hot and clammy; perspiration rancid; bowels open, but not freely purged.

> Rep. Calomel, ℈ss.; Opii, gr. ij. omni nocte et mane.
> Haust. Sennæ c. Tinct. Jalapæ, ʒi. meridie quotidie.
> (Let him be kept comfortably cool.)

22d.—The wrist and fore-arm of the left side free from disease. The right hand and arm still puffy and painful. None of the synovial membranes or bursæ are distended with fluid. Pulse seventy-two, full and soft; tongue red and clean; skin hot and dry; bowels open.

Rep. Calomel et Opium omni nocte.
Haust. Sennæ c. Tinct. Jalapæ omni mane.

24th.—No complaint, except slight stiffness of the left foot and leg. No fever. Mouth slightly affected by the calomel.

Omitt. Pilulæ Calomel et Opii.

Sumat Pulv. Ipecac. c. ℈ss. omni nocte.

Haustus Sennæ alterna quaque mane.

Haustus Cinch. bis die.

Garg. Aluminis.

June 10th.—Has been gradually convalescent since last report: complains only of some remains of pain in the right shoulder.

II. October 13th, 1825.—Joseph Edwards, æt. thirty-four, mason.

Complains of rheumatic pains and diffused swelling of the limbs, particularly in the neighbourhood of the wrists and shoulders. The pains are not much altered by variations of temperature. Pulse 120, full; skin hot; tongue white; bowels costive; urine high coloured. Has had slight pains of limbs for three weeks, and nine days ago was seized with severe pains and swellings of the feet, ankles, and the neighbourhood of the knees. The disease, in a day or two afterwards, left these parts, and attacked the present seats of pain and swelling.

Calomel, ℈ss.; Opii, gr. ij. omni nocte et mane.

Haustus Sennæ meridie quotidie.

Diæta sit parcissima.

20th.—Mouth slightly sore. Pains relieved.

Opii, gr. ij. bis die. Omisso Calomel.
Haustus Sennæ omni manè.

22d.—Pains and swellings quite gone. Pulse eighty, full and soft; skin cool; tongue furred in centre, clean at edges; bowels open. He sleeps ill.

Repet. medicamenta.

27th.—Cured.

He took the haustus cinchonæ for the last three days, and had a blister applied for a slight pain in the right shoulder.

When patients apply soon after the commencement of the attack, the disease sometimes yields very speedily to this method of treatment: the following may be regarded as illustrations.

