# Errata

**Published:** 1826-08

**Authors:** 


					P. 12, Jin* 3# for "bass," read "bags."
P. 13, second prescription, for
a Item a quaqae mane,** read ''alternis diebus mane."

				

